# Antimicrobial peptides: Defending the mucosal epithelial barrier

**DOI:** 10.3389/froh.2022.958480

**Published:** 2022-08-01

**Authors:** Karen F. Johnstone, Mark C. Herzberg

**Affiliations:** Department of Diagnostic and Biological Sciences, School of Dentistry, University of Minnesota, Minneapolis, MN, United States

**Keywords:** epithelium, antimicrobial peptides/proteins, calprotectin, defensins, LL-37, health, disease

## Abstract

The recent epidemic caused by aerosolized SARS-CoV-2 virus illustrates the importance and vulnerability of the mucosal epithelial barrier against infection. Antimicrobial proteins and peptides (AMPs) are key to the epithelial barrier, providing immunity against microbes. In primitive life forms, AMPs protect the integument and the gut against pathogenic microbes. AMPs have also evolved in humans and other mammals to enhance newer, complex innate and adaptive immunity to favor the persistence of commensals over pathogenic microbes. The canonical AMPs are helictical peptides that form lethal pores in microbial membranes. In higher life forms, this type of AMP is exemplified by the defensin family of AMPs. In epithelial tissues, defensins, and calprotectin (complex of S100A8 and S100A9) have evolved to work cooperatively. The mechanisms of action differ. Unlike defensins, calprotectin sequesters essential trace metals from microbes, which inhibits growth. This review focuses on defensins and calprotectin as AMPs that appear to work cooperatively to fortify the epithelial barrier against infection. The antimicrobial spectrum is broad with overlap between the two AMPs. In mice, experimental models highlight the contribution of both AMPs to candidiasis as a fungal infection and periodontitis resulting from bacterial dysbiosis. These AMPs appear to contribute to innate immunity in humans, protecting the commensal microflora and restricting the emergence of pathobionts and pathogens. A striking example in human innate immunity is that elevated serum calprotectin protects against neonatal sepsis. Calprotectin is also remarkable because of functional differences when localized in epithelial and neutrophil cytoplasm or released into the extracellular environment. In the cytoplasm, calprotectin appears to protect against invasive pathogens. Extracellularly, calprotectin can engage pathogen-recognition receptors to activate innate immune and proinflammatory mechanisms. In inflamed epithelial and other tissue spaces, calprotectin, DNA, and histones are released from degranulated neutrophils to form insoluble antimicrobial barriers termed neutrophil extracellular traps. Hence, calprotectin and other AMPs use several strategies to provide microbial control and stimulate innate immunity.

## Introduction

Encounters with infectious agents or tissue injury cause inflammation, the initial response for tissue repair and innate immune defense [1]. The recent epidemic caused by SARS-CoV-2 resulted in millions of deaths worldwide in only 2 years [2–6]. Infections caused by this airborne virus [7, 8] reveal the vulnerability of our mucosal epithelial barriers against infections. Among the oldest known immune defense molecules, antimicrobial proteins/peptides (AMPs) are ubiquitous through evolution and across species. The first identified AMPs in *Precambrian* protozoans date to 1,000 million years ago [9]. Most prominent at the epithelial barrier where most infections occur, AMPs are crucial to host defense, and about 45 AMPs have been identified in human saliva and in the oral environment [10]. To understand the contribution to epithelial barrier defense, AMP function in higher animals must be distinguished from adaptive and other innate immune mechanisms. Some AMPs show broad-spectrum activity across phyla, whereas others are more specific. Even in the face of posited resistance mechanisms, therapeutic strategies are in development to fortify antimicrobial defense at the epithelial barrier. Indeed, translation of knowledge of the structure and function of AMPs may herald a post-antibiotic era.

### The epithelial barrier in resistance to microbial infection

Barrier protection against infection by exogenous bacteria, fungi, and viruses is essential for survival. Formed by epithelia, the barrier physically partitions the underlying connective tissues from the external environment [11]. Barrier breaches are defended by host adaptive and innate immune mechanisms.

Innate responses are activated by the engagement of signaling pathways including Toll, Immune deficiency (IMD), and Janus Kinase and Signal Transducer and Activator of Transcription (JAK/STAT) [12]. These three pathways are found in virtually all life forms, both ancient and contemporary. For example, recognition and responses to pathogen-associated molecular patterns (PAMPs) in insects involve signaling pathways that are highly homologous to humans [12]. Crosstalk between the Toll and IMD signaling cascades induce the production of AMPs. The three pathways, however, partition antimicrobial responsibility. JAK/STAT is activated by infection or sepsis [13], producing the downstream effectors cytokines and stress response proteins [14]. The Toll family of receptors distinguish and signal in response to PAMPs originating from Gram-positive and Gram-negative bacteria, fungi, viruses [15, 16], and damage/danger-associated molecular patterns (DAMPs) that are released from injured tissues [16–18]. The IMD effects humoral immunity against Gram-negative bacteria and fungi [14], illustrating a redundancy in primitive immune function. Indeed, immunity in lower life forms reflects low specificity and broad, overlapping recognition and signaling responses to pathogens.

Functioning to protect epithelial barriers, the effectors of innate immune functions in the host span phylogeny and evolutionary time. Produced as part of the transcriptional response to engagement of PAMPs, many AMPs are highly conserved in invertebrates and serve as the primary humoral response [19]. In contrast, a minimally competent cell-mediated immune response is provided by hemocytes. In lower life forms, the AMP-mediated innate immune response is central to resistance against pathogenic microbes and survival.

To illustrate the scope of AMP production across species, *Drosophila* [19], the domesticated silk moth, *Bombyx mori* [20], and *Rhynchophorus ferrugineus* (red palm weevil) [21] use the Spätzle-mediated activation of the Toll pathway to upregulate genes for multiple AMPs. AMPs produced in response to activation of the Toll-Spz pathway also provide antimicrobial immunity in *Manduca sexta* [22], *Antheraea pernyi* [23], mosquito species [24], and shrimps [25]. Using genome wide analysis, Manduca potentially express 86 different AMPs [26]. Remarkably, Manduca also show intergenerational immunity, whereby offspring show increased AMP production after mothers were infected with *Serratia marcescans* [27]. The upregulated AMPs provide immunity against many common bacterial and fungal pathogens.

In the mealworm beetle, *Tenebrio molitor*, a frank breach in the cuticle epithelium accompanied by microbial challenge triggers local epithelial production of AMPs such as cecropins [28, 29] and melanin to thwart parasitic infection of plants and attenuate parasites and pathogens, including fungi [30]. Mealworms, then, illustrate that the cuticular epithelium can deploy different AMPs, eliciting somewhat specialized antimicrobial responses as an infection becomes more invasive.

More closely related to vertebrates, Tunicates including *Ascidiae*, inhabit marine environments, where they encounter infectious agents such as viruses, bacteria, and fungi in the pharynx [31]. The pharynx functions for breathing and food collection and is the primary immune organ [31]. Reflecting an innate response of greater sophistication, *Ciona robusta* utilize hemocytes in the hemolymph to initiate inflammation [32]. Unique to these invertebrates, *C. robusta* express innate immune receptors including secreted immunoglobulin Variable-region containing Chitin Binding Proteins (CrVCBPs) early in the response [32]. CrVCBPs behave as antibodies of restricted specificity enabling a response to lipopolysaccharides that are common to their colonizing microbes. AMPs produced by Tunicates act as the first line of epithelial defense against pathogens including bacteria, fungi, viruses, and parasites [33].

This discussion illustrates that the lowest life forms express AMPs, which enable organisms to resist human pathogens such as *Staphylococcus aureus*. In the skin of a frog, the antimicrobial products of different genes can work synergistically in the same tissue. The granular glands of the splendid leaf frog, *Cruziohyla calcarifer*, produce cruzioceptins which are 21–23 residue alpha helical cationic peptides with antimicrobial activity against *E. coli, S. aureus*, and *C. albicans* [34]. In the Australian tree frog, the three AMPs from the skin (e.g., aurein 1.2, maculatin 1.1, and caerin 1.1) function synergistically against Gram-negative *Escherichia coli* and Gram-positive *Staphylococcus aureus* [35]. Expressed by species through evolution, two or more unique AMPs work synergistically to provide overarching defense against infection by exogenous organisms and control or limit the growth of commensal species [9]. In lower animals, AMPs function typically in the absence of adaptive immunity. Virtually every life form employs AMPs to provide anti-bacterial and anti-fungal defense.

Since most human infections require that microbes breach the epithelial barrier, epithelial cells have evolved to contribute to resistance to infection by providing robust local immunity. During oral mucosal health, constitutive expression of AMPs likely serves to control the overgrowth of commensal species, the emergence of pathobionts, and dysbiosis. Antimicrobial proteins are expressed by epithelial cells from tissues ranging from the gingiva [36–38] to the cornea of the eye [39] and to the Paneth cells of the intestines [40]. These epithelial AMPs confer protection against infection by bacteria [41–43], protozoa [44], fungi [45, 46], and viruses [47, 48]. By studying interactions between AMPs and the transcriptional response of bacterial cells and applying artificial intelligence algorithms, AMPs could be engineered in the future to overcome microbial resistance mechanisms.

### Microbes drive the development of innate oral immunity

The pressure of early colonizing commensal bacteria appears to drive epithelial development, maturation, and AMP expression. Using reconstituted human gingiva, incubation with the prominent salivary organisms *Granulicatella, Veillonella*, and *Streptococcus* promotes keratinocyte proliferation, thickening, and greater organization of the epithelial layer [49]. Epithelial maturation was accompanied by increased expression of several key regulated AMPs including elafin, hBD2, hBD3, adrenomedullin, and cathelicidin (LL-37), and secretion of antimicrobial AMPs (e.g., IL-6, CXCL8, CXCL1, CCL2). Except for hBD1, other AMPs were not studied. These *in vitro* data suggest that in the absence of leukocytes, the commensal microbiota drives the development of the epithelial barrier against infection.

The role of the oral microbiota in driving mucosal maturation including innate immunity is supported by *in vivo* studies in mice. In mouse neonates, microbes colonize the oral mucosa at high levels. The prenatal oral mucosa expresses the chemotactic cytokine, IL-17, which apparently recruited neutrophils since these phagocytes were not seen in *Il-17*^−/−^ mice [50]. IL-17 and neutrophils virtually disappear by 4-week of age in the buccal mucosa, while persisting in the gingiva and the junctional epithelium into adulthood. The presence of IL-17 and neutrophils was directly associated with the presence of γδT cells. After weaning, the density of oral mucosal microorganisms reduces to adult levels, and the *Streptococcaceae* and other genuses outgrow the *Pasteurellaceae* in mice. The microbiota drive the maturation of the oral epithelium including loss of permeability, lower turnover rate, and increased expression of AMPs as marked by CRAMP, the murine analog of human LL-37, and β-defensins 4 and 14. Postnatally, the production of AMPs in saliva reduces the oral microbial load [50]. Microbial colonization is essential for normal mucosal maturation and the development of local innate immunity in this *in vivo* model.

In epithelia, the control of AMP expression involves pathogen-recognition receptors (PRRs) that are conserved in insects, worms, and in humans and other mammals [9]. By engaging a pathogen-associated molecular pattern (PAMP) representing virulence factors on microbes, PRRs activate the mTOR and NF-kB pathways to promote expression of many immune response genes including AMPs. The AMPs produced in higher life forms are generally conserved from lower life forms [9]. AMPs such as hCAP-18, which is cleaved to release the bioactive peptide LL-37, and calprotectin (e.g., complexed S100A8 and S100A9; S100A8/A9) are found in humans and other mammals and are inducible in epithelia [51–53]. The more evolutionarily modern epithelial AMPS such as calprotectin present new antimicrobial mechanisms and function by sequestering essential trace metals from microbes [54, 55], whereas more ancient AMPs form pores in microbial membranes [56]. Given the very different modes of action, the two mechanisms of AMP activity can function synergistically [57–59].

In humans and other mammals, AMPs in the extracellular milieu protect epithelia against tissue-invasive microbes. In this environment, insoluble neutrophil extracellular traps (NETs) form after dead and dying cells release AMPs, DNA, and histones [60–62]. These structures trap proximal microbes and use the bound AMPs to kill or prevent bacterial and fungal growth. Within viable resting cells, AMPs, including defensins and cathelicidins, typically localize in cytoplasmic granules [63–66] and calprotectin in the cytoplasm [67, 68].

Within cells, AMPs in granules and in the cytoplasm (e.g., calprotectin) contribute to antimicrobial defense. During microbial invasion or phagocytosis, AMP-containing granules fuse with phagosomes or endosomes where invasive microbes localize [69–72]. After release into the endosome or phagolysosome, AMPs cooperate with other antimicrobial mechanisms, including the production of reactive oxygen species to inactivate and kill intracellular pathogens.

AMPs found exclusively in the cytoplasm of viable cells include calprotectin and LL-37 and appear to protect cells from invasive pathogens that escape endosomes and phagosomes. Some invasive pathogens like *Listeria monocytogenes* use listeriolysin O (LLO) and lipases to digest the endosome and phagosome membranes and escape into the cytoplasm [73–75]. Cells have apparently co-evolved to protect against microbial invasion into the cytoplasm. Human α-defensins block LLO-dependent membrane pore formation, release of LLO from the bacteria, and intracellular multiplication [76]. *Listeria* counter by activating type I interferons, which suppress phagosome maturation and proteolysis of LLO and facilitate escape and spread of infection [77]. As *Listeria* exits the phagosome, however, calprotectin in the cytoplasm of epithelial cells and neutrophils limits intracellular bacterial growth [68]. The antimicrobial activity of calprotectin against *Listeria* is enhanced by ubiquitination of InlC, which stabilizes the S100A9 subunit of calprotectin and causes an increase in ROS production by neutrophils in mice [78]. At 2 h in the cytoplasm, growth of *Listeria* and *Salmonella typhimurium* are inhibited by calprotectin and LL-37 [68, 79, 80]. At the same time, up to 10–12% of epithelial cells show LLO-dependent calprotectin mobilization; mobilized calprotectin complexes with polymerized cytoplasmic microtubules [68]. When complexed with microtubules, anti-*Listeria* activity is subverted. Listeria also causes epithelial cells to upregulate IL-1a, which signals the IL-1 receptor in an autocrine loop to upregulate calprotectin [81–83]. Cytoplasmic AMPs, therefore, largely function to control microbes within cells or resist intracellular invasion.

## Spectrum of AMP activity across microbial kingdoms

**Figure 1:**
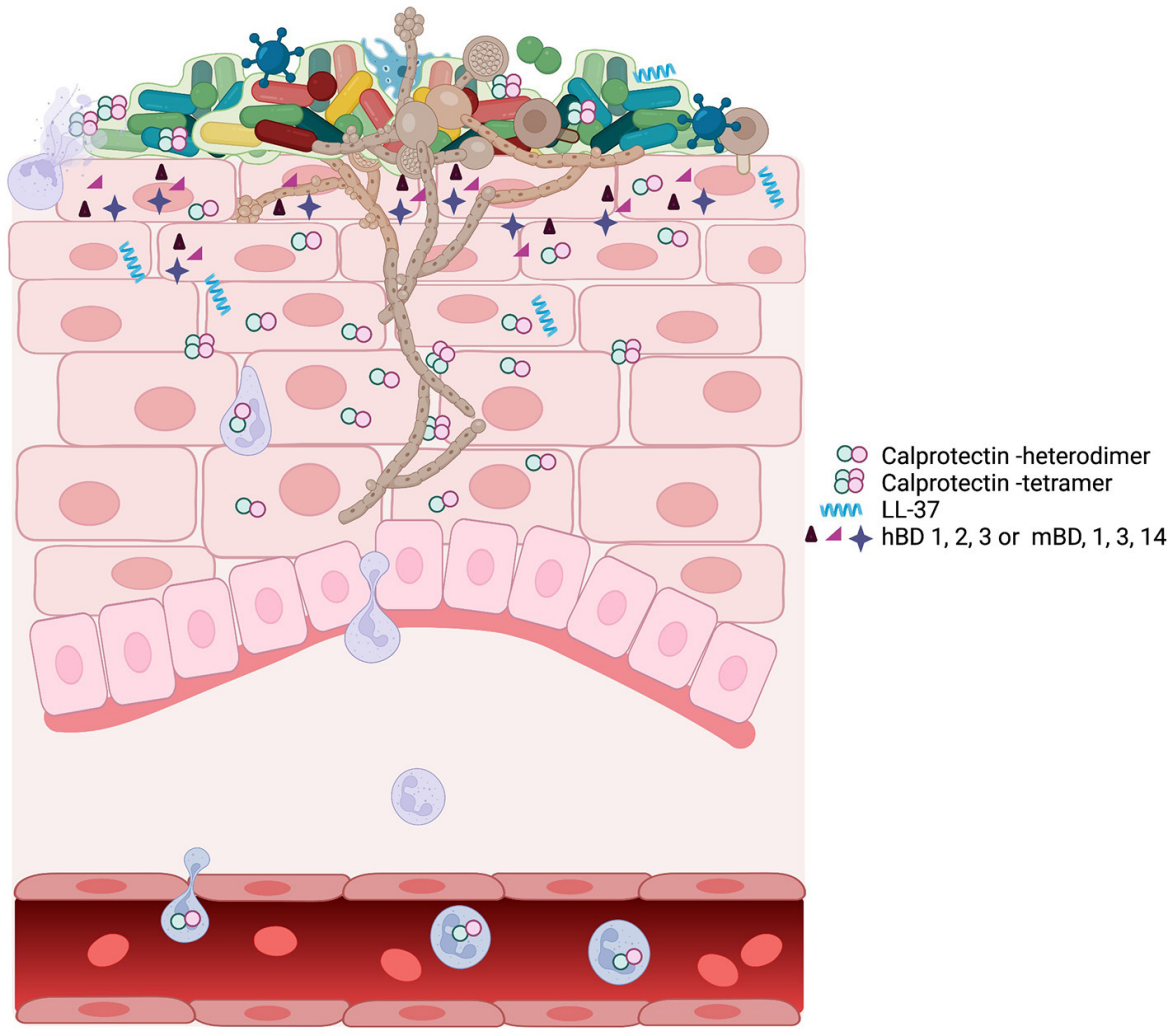
A schematic view of oropharyngeal candidiasis showing the complex participants in the infection. The oral microbiome associated with candidiasis is different from the oral microbiome in health [86]. The outgrowth of *C. albicans* hyphae may be facilitated by or drive the changes associated with the dysbiotic microbiome. *C. albicans* in a dysbiotic community leads to inflammation and upregulation of many AMPs including calprotectin, LL-37, and defensins. At the site of infection, the expression of AMPs, the dysbiotic microbiome, and the outgrowth of *C. albicans* can individually or collectively signal for infiltration by immune cells, including neutrophils, macrophage, and T cells. Neutrophils and macrophage phagocytose Candida. Neutrophil degranulation provides the components for neutrophil extracellular traps, which contain the spread of Candida amid high concentrations of AMP calprotectin [67].

Many species of bacteria and fungi are cell invasive, and cytoplasmic AMPs limit the extent of intracellular invasion. Common fungi that can be identified in the oral epithelial tissues include *Candida, Malassezia, Cryptococcus*, and *Trichoderma* spp. [84]. *Candida albicans* and other fungal species persist as low abundance commensals which emerge prominently in the immunocompromised [85] (Figure 1). These fungi appear to be kept in check by neighboring mucosal microbiota and may overgrow because of interkingdom changes centering on the oral microbiome [87]. Antifungal proteins of the oral cavity include the histatins [88], α- and β-defensins [89], and calprotectin [90, 91]. In saliva, fungal growth is controlled by several known antimicrobial proteins including CCL28 [92], histatin 5 [93], the cystatins [94], and MUC7 [95]. Proximal to the mucosal surfaces, pathogenic *Candida albicans*, the most common pathogenic fungus in the oral cavity, secrete candidalysin from hyphae [96]. Candidalysin activates oral epithelial cells *via* EGFR to produce a robust antifungal response [97]. The epithelial response includes the release of anti-fungal hBD2 and hBD3, LL37, S100A8, and ATP, which appears to transactivate neighboring epithelial cells. Released S100 proteins and defensins from epithelial cells into the tissues serve to recruit neutrophils containing a potent redundant armamentarium of AMPs [97]. The antifungal responses of epithelial cells are made more robust by epithelial IL-17 signaling, which increases expression of β-defensin 3 and thwarts oropharyngeal candidiasis in mouse models [98]. Similarly, deficiency in mouse β-defensin-1 results in IL-17 deficiency and mucosal candidiasis upon infection with *C. albicans* [99] (Figure 1).

Viruses that typically infect oral epithelial tissues include the herpesviruses: Herpes Simplex type 1 (HSV-1), Epstein-Barr virus (EBV), cytomegalovirus (CMV), and Kaposi's sarcoma-associated herpesvirus (KSHV) [100]. Antimicrobial peptides appear to mitigate infections by acting directly on herpes simplex type 1 [101–104], SARS-CoV-2 [105], and KSHV [48]. The AMP LL-37 appears to inhibit infection by KSHV [48], whereas α-defensin-derived peptide HD5 suppresses infection by CMV [106], and lactoferrin attenuates HSV-1 [107]. Generally acquired by sexual activity, human papilloma virus (HPV) is thwarted by α-defensin 5 [108] and theta-defensins [109], although clearance is multifactorial [110]. To produce a new generation of therapeutic agents, naturally occurring AMPs are being packaged [111] and re-engineered [112] to optimize direct antiviral activity.

Although not considered an oral virus, HIV-1 interactions with oral mucosal epithelium result in non-permissive infection and the virus does not replicate in the oral mucosa [113]. In the oral tissues, AMPs could contribute to local viral restrictions that curtail replication since the anatomically similar genital mucosa do become infected. Upon oral exposures, HIV-1 must translocate across mucosal epithelia to infect subepithelial CD4 T cells, which may be facilitated by the induction of epithelial-mesenchymal transition [114]. Translocation of HIV-1 may also be enabled by a co-infection with the periodontal pathogen, *P. gingivalis* [113, 115–117]. Rapid uptake by epithelial cells enables HIV-1 to escape inactivation by human saliva [118] without appearing to permit productive infection of keratinocytes [113]. Among the restrictions of HIV-1 replication in mucosal epithelial cells and vaginal fluid are cationic antimicrobial peptides including calprotectin, hBD-1 and 2, and the secretory leukocyte protease inhibitor (SLPI) [119]. Although LL-37 appears to inhibit HIV-1 replication in peripheral blood mononuclear cells [120, 121], this AMP enhances HIV-1 infection in intraepithelial Langerhans cells [122]. The contribution of AMPs to resist productive HIV-1 infection in the oral mucosa has not been definitively established.

Recently SARS-CoV-2 has been shown to infect salivary glands, which express the cognate ACE2 receptor [123]. In the salivary glands, SARS-CoV-2 harbors, replicates, and is subsequently released in saliva [124, 125]. Surprisingly, SARS-CoV-2 infections are mitigated by antimicrobial peptides (as is HSV-1) [105]. Binding of hBD-2 blocks ACE2-mediated entry of SARS-CoV-2 into target epithelial cells [126].

Tissue-specific expression of AMPs could explain tropisms that govern the mucosal sites of viral infections. Consider that influenza type A virus (IAV) infection is confined to the upper respiratory tract although it is contiguous with but does not infect the oral mucosa. The anatomic site sequestration is somewhat paradoxical. The major AMPs in the salivary film coating the oral mucous membranes include HNPs, hCAP18/LL-37, hBDs [127], and salivary mucins [128]. Studies in primates suggest that many more AMPs are expressed in the microbially responsive gingival epithelium [129]. Indeed, human saliva also contains the antifungal histatins [93, 130] and the antifungal/antibacterial AMP, calprotectin [131]. The known antiviral proteins/peptides are high molecular mass glycoproteins [132]. Similar to the oral content, the predominant AMPs produced at the mucosal surfaces of the upper airways are neutrophil α-defensins/HNPs, HBDs, LL-37, sPLA2-IIA [133], and calprotectin [134]. Perhaps critically, upon infection the airway secretions become acidified which mitigates the effectiveness of the AMPs. The saliva and oral mucosal surfaces contain mechanisms that limit acidification [135], with specialized microbial domes that appear to contain acid that demineralizes the tooth structure [136].

We speculate that acidification of the airway fluids during infection mitigates the effectiveness of the AMPs, facilitating common co- or secondary-infection with *Streptococcus pneumoniae* in the respiratory epithelium caused by influenza [137]. Proximal IAV infection would appear to be stimulated by the neuraminidase-expressing streptococci, which include certain oral mitis group streptococci. Resulting interferon signaling increases the production of mucins [138], contributing to the release and spread from epithelial cells [139, 140]. The streptococci would be expected to be sensitive to the local AMPs if the secretions were not acidified [133]. Furthermore, IAV replication in airway epithelial cells appears to be stimulated by uncharacterized products of *S. oralis* and *S. mitis*, among Mitis group streptococci [141]. Although Mitis group streptococci are the most frequently recovered bacteria from bronchoalveolar lavage during aspiration pneumonia [142, 143], the seeming resistance of oral tissues to IAV infection is surprising. During IAV infection, both type I interferon production [138] and TGF-β-mediated expression of host cell integrin receptors [144] promote bacterial co-infection. Signaling through the IL-17 receptor upregulates anti-fungal AMPs [98, 145]. Unlike pulmonary fluids, the buffering capacity of saliva may allow for sustained AMP activity and suppression of intruding *S. pneumoniae* and provide a mechanistic explanation for the resistance of the oral cavity to IAV infection.

## Specific contribution of AMPs to antimicrobial defense

In higher animals, the efficacy of AMPs has been determined by isolating and purifying the respective proteins/peptides followed by *in vitro* testing for antimicrobial function. In the host, however, the contribution of AMPs to broad antimicrobial defense is less clear because of the complexity and seeming functional redundancies of the innate and adaptive immune responses. And indeed, the AMPs can signal and modify adaptive immune responses [146–149], thereby providing points of convergence with innate immunity.

To resolve the functions of AMPs with greater clarity, animal models are useful. Animal models can facilitate understanding of modes of action, including knowledge of how interactions with inflammatory responses and adaptive immunity affect the composition of the microbiome. The microbiome can profoundly affect host health. Shifts in the microbiome can lead to dysbiosis and the emergence of pathobionts or to the acquisition of pathogens that are related to many diseases, including metabolic and neurologic disorders. AMPs that are constitutively expressed or upregulated during inflammation can contribute to homeostasis of the microbiome.

## Defensins function as pore-forming antimicrobials in humans and other mammals

Defensins are cationic peptides that bind and form pores in bacterial and fungal membranes causing increasing membrane permeability and cell death [150]. A query of the genome sequences of 29 vertebrate species revealed that humans have genes for 31 β-defensin peptides, mice have 38, rats have 41, and cattle have 42 [151]. Only a few of the gene protein products have been evaluated for antimicrobial activity and immune function. Since studies analyze one family member at a time, the seeming redundancy in defensins (and other AMPs) leaves open the question of whether AMPs synergize or compliment their activities. The effectiveness of the defensins may be limited by environmental factors including inactivation by high salt concentration and degradation by protease activity. Clearly, expression and AMP effectiveness can differ.

There are two main families of defensins found in saliva [152, 153]. The α-defensins (HNP1, HNP2, HNP3, HNP4) are made by neutrophils [154] and are released into the saliva during infections and inflammation. β-defensins (hBD1, hBD2, hBD3, hBD4) are synthesized by mucosal epithelial cells and can be found at the mucosal surface in gingival crevicular fluid and saliva [49]. Although regulated in response to viral agents [47, 155], human β-defensin 1 (HBD1) is generally constitutively expressed [156], whereas HBD2 and HBD3 are induced by microbial insults and pro-inflammatory cytokines in various mucosal epithelial tissues [49].

Oral candidiasis or thrush is common in denture wearers, in people with salivary gland defects, and in the immunocompromised. In a model of oral infection by *Candida albicans*, wild type mice (DEFB1^+/+^) showed an influx of neutrophils at the sites of intraepithelial infection and adjacent to the fungal hyphae [99] (Figure 1). Mice deficient in the constitutively expressed mouse β-defensin-1 (DEFB1^−/−^) showed more sparse neutrophil infiltrates and increased fungal burdens at days 3 and 7 after inoculation [99]. The increased susceptibility to infection by *C. albicans* in DEFB1^−/−^ mice may also be explained by reduced induction of mucosal AMPs including cathelicidin antimicrobial peptides (CAMP), LL-37, mBD2, and mBD4. The risk of infection may also increase if levels of calprotectin, chemokine CXCL1, and cytokines Il-17A, IL-17F, Il-6, and IL-1β are also reduced. In the DEFB1^−/−^ mice, which are also deficient in CAMP, mouse β-defensin-1 appears to contribute to persistent antifungal activity and regulate host inflammatory responses [99]. Neutrophils and macrophages from the DEFB1^−/−^ and wild type mice were similar in *in vitro* neutrophil killing assays and IL-1β release. Mouse genotype-specific differences in infection by *C. albicans* could also be attributed to competition with co-colonizing microbiota and the responses of T cells and other immune response elements.

In DEFB1^−/−^ mice, IL-17 cytokines are associated with susceptibility to oral Candida infection [98]. In wild type mice, IL-17 signaling recruits neutrophils to the oral epithelium which can clear infections [50]. The IL-17 receptor pathway and the expression of the IL-17 receptor on surface mucosal epithelial cells (keratin 13 expressing) are also crucial to upregulating the AMPs that aid in controlling the Candida infection [98]. IL-17R plays a part in regulating the oral epithelial cell response to infection by Candida. When challenged with Candida, the susceptibility to infection of Il17ra^−/−^ and Il17r conditional deletion (Il17ra^Δ*K*13^) mice was similar and associated with induction of the AMP, murine β-defensin-3 [157]. DEFB1 expression was also similar in control mice and those with oral (local) or complete IL-17 receptor deficiency. The IL-17 receptor controls the expression of several antimicrobial peptides including S100A8, S100A9, lipocalin 2, and mBD3 [145]. DEFB3^−/−^ mice had the same susceptibility to oral candidiasis as the Il17ra^−/−^ mice, highlighting the importance of IL-17 receptor and murine β-defensin-3 in antifungal immunity by immunocompetent mice [98].

The different dependencies of mBD1 (DEFB1^−/−^ mice) [99] and mBD3 (Il17ra^−/−^ mice) [98] on mitigating oropharyngeal candidiasis is interesting. In both models, the expression of non-inducible mBD1 is expected, but mBD3 is upregulated, however, by IL-17 receptor signaling in association with lipocalin-2 and calprotectin [98]. IL-17 promotes a 10–40-fold increase in calprotectin expression in keratinocytes *in vitro* and in mouse oropharyngeal candidiasis [158]; inflammation caused by experimental periodontitis also upregulates calprotectin [159]. The diminished antifungal activity in the Il17ra^−/−^ mice may reflect a loss of synergy between several different AMPs including mBD3, lipocalin-2, and calprotectin. Indeed, purified calprotectin with lactoferrin completely inhibits *C. albicans in vitro*. Calprotectin and lactoferrin can both be released from neutrophils suggesting that they could also work cooperatively at sites of inflammation to clear *C. albicans* [160].

In the oral cavity, β-defensins may also protect against bacterial infections. Using a mouse model, a *P. gingivalis*-soaked ligature was used to induce experimental periodontitis, and HBD3 was applied to the periodontal pocket [161]. After three applications of HBD3, ligated sites showed reduced osteoclast and alveolar bone loss, and markers of periodontitis: MMP-9, TNF-α and IL-6. Incubation of HBD3 with a macrophage cell line attenuated polarization into a proinflammatory M1 phenotype. While it is unclear whether the human BD3 affected the expression of mouse β-defensins, calprotectin, or other synergistic oral AMPs, application of HBD3 appears to have therapeutic potential.

## Calprotectin as a location-dependent AMP

### Extracellular calprotectin

**Figure 2:**
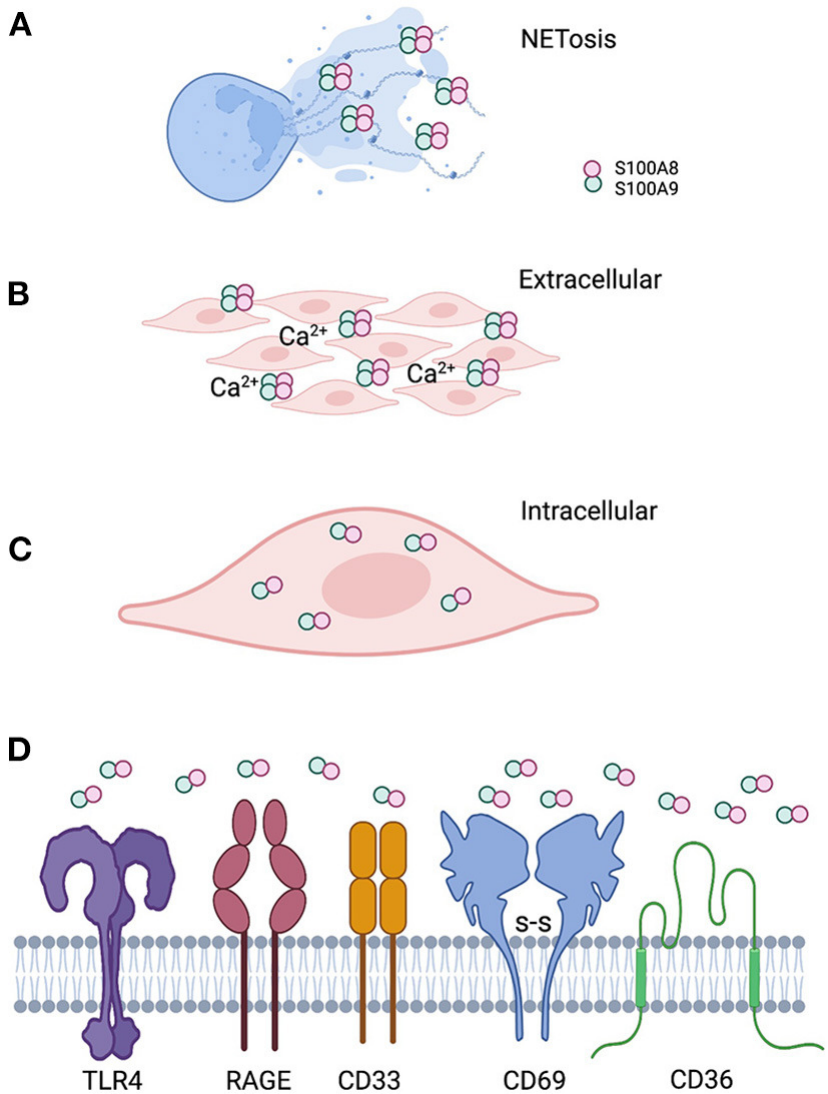
Localization of calprotectin at the epithelial barrier specifies function. **(A)** Calprotectin released from degranulated neutrophils and epithelial cells complex with DNA and histones to form antimicrobial neutrophil extracellular traps. **(B)** When calprotectin is released from cells in a high calcium concentration inflammatory environment, the soluble AMP forms heterotetramers, providing increased affinity for trace metal divalent cations. Successful sequestration of the trace metals from microbes results in reduced growth and “nutritional immunity.” **(C)** Calprotectin localized within the cytoplasm of epithelial cells or neutrophils appear to protect against invasive microbes that seek to reside intracellularly as part of their life cycle. Whether intracytoplasmic calprotectin with AMP activity exists as monomers, heterodimers. or heterotetramers, assuming increased cytoplasmic calcium upon release from intracellular stores, remains to be studied. **(D)** Released calprotectin can engage a range of receptors on cells in the inflammatory environment. Engagement with specific receptors link this AMP with a range of innate cellular immune responses.

Calprotectin, a heterodimer of S100A8 and S100A9 (S100A8/A9), is a multifunctional AMP expressed in the cytoplasm of mucosal epithelial cells and neutrophils. In neutrophils, calprotectin comprises about 45% of the total cytosolic protein. Functionally prominent in the oral cavity, calprotectin is also found in the saliva [162, 163] and gingival crevicular fluid [164–167]. During infection and inflammation, the function of calprotectin can change in response to the local conditions (Figure 2).

Calprotectin has no signal sequence and is not secreted *via* the classical endoplasmic reticulum-Golgi pathway. At the mucosal surface, calprotectin enters the extracellular space when released by activated monocytes [168] or neutrophils [67]. Extracellular calprotectin can also undergo oxidation [169], thereby increasing its vulnerability to proteolysis and fragmentation into smaller peptides of yet unknown biological activity [170]. In the extracellular space, calprotectin is available to bind receptors (Table 1, Figure 2), assemble heterodimers and other oligomeric forms [189], and sequester metal ions [190]. Soluble calprotectin may be recognized as a damage-associated molecular pattern (DAMP) or alarmin to activate neutrophils or function as an antimicrobial protein to suppress microbial growth [191]. Antimicrobial activity is increased locally when calprotectin incorporates into neutrophil extracellular traps (NETs) [191].

**Table 1:** Cell receptors for calprotectin.

Calprotectin receptor^*^	**Putative function**	**Tissue/cell**	**References**
TLR4 and RAGE	Secretion of proinflammatory cytokines	BV-2 microglial cells	[171]
	Upregulation of MDSCs; inhibition of dendritic cell differentiation	Myeloid-derived suppressor cells; dendritic cells	[172, 173]
TLR4	Regulate the inflammatory cascade during sepsis	Phagocytes	[174]
	Regulates inflammation during virus infection	Lung	[175]
	Costimulatory enhancer of inflammation to upregulate IL-17 *via* ligation of TLR4 on CD8+Tcells	CD8+ T cells	[176]
	Induces IL-6 and MCP-1 *via* TLR4 signaling *via* MAPK and NF-κB, resulting in the progression of periodontitis	Fibroblasts	[177]
RAGE(s100A9)	Induces NET formation in humans	Neutrophils	[178]
	Induces NET formation in mice	Neutrophils	[179]
RAGE	Decreased cardiac contractility	cardiomyocytes	[180]
	Promotes inflammatory microenvironment required for tumor development	Immune cells in the epidermis	[181]
	Enhanced cytotoxic activity of NK cells	S100A8/A9 expressing pancreatic tumor cells/Natural killer cells	[182]
	Induces chemotaxis of neutrophils and secretion of proinflammatory cytokines		[183]
CD36	Fatty acid uptake by endothelial cells	Endothelial cells	[184]
CD36(S100A9)	MRP-14 binding to platelet CD36 regulates arterial thrombosis	Platelets	[185]
CD33(S100A9)	Expansion of MDSC perturbs hematopoiesis and contributes to the development of myelodysplastic syndromes	Myeloid-Derived suppressor cells (MDSCs)	[18]
CD69	T-cell differentiation	T cells	[186]
TLR3 (S100A9)	regulator of TLR3 signaling; functions during pre-TLR3 activation by enabling maturation of TLR3 containing early endosomes into late endosomes	Bone marrow–derived macrophages	[187]
TLR2	TLR2/S100A9/CXCL-2 signaling network for neutrophil recruitment	Neutrophils	[188]

* *The receptors respond experimentally to calprotectin or the indicated subunit. In general, the multimeric form of S100A8/A9 is not reported*.

NETs are an insoluble mesh formed primarily from the released granular and cytoplasmic contents of dead and dying neutrophils, which include DNA, histones, and calprotectin among other AMPs [192]. Calprotectin in NETs may be essential to clear infections in the tissues as observed in a mouse model of *Candida albicans* infection [67]. When complexed in NETs, calprotectin can activate neutrophils by upregulating CD11b and increasing cellular adhesion [191]. S100A9 released from the neutrophil can bind the neutrophil RAGE receptor and potentiate further release of NETs [178].

In the extracellular space, calprotectin sequesters nutritional metal ions which promotes antimicrobial “nutritional immunity” [190, 193, 194]. Each S100 subunit contains two Ca^2+^ binding sites [195, 196]. In the absence of Ca^2+^ ions, S100A8/A9 exists as a heterodimer, and upon Ca^2+^ binding, the two heterodimers self-associate to form a heterotetramer [189, 196]. Hetero-multimerization can also be promoted by oxidative cross-linking during inflammation [197]. In comparison to its human ortholog, murine calprotectin requires 10-fold more Ca^2+^ equivalents to form a heterotetramer [198]. Each heterodimer contains two distinct transition-metal-binding sites. When compared with the heterodimer, calcium-induced tetramers show enhanced binding affinity for Mn, Fe, Ni, Cu, and Zn and increased protease stability [189, 196]. By sequestering transition metals, calprotectin heterotetramers starve microbes that infect the surrounding inflammatory tissue. Calprotectin heterotetramers also lose DAMP activity because this multimer shows reduced access to the TLR4/MD2-binding site, which mitigates a broader inflammatory response [199]. By reducing the inflammatory response, off-target tissue damage is minimized during clearance of the infection [200–203].

Some bacterial pathogens have evolved to thwart nutritional immunity. To compete with calprotectin and commensal species for nutritional metal ions, pathogens utilize and can increase avidity of metal ion transporters [204, 205]. By sequestering Mn^++^, for example, Salmonella resists killing after phagocytosis by inhibiting neutrophil Mn^++^-dependent enzymes that detoxify reactive oxygen species [205].

Some Gram-positive species may benefit when calprotectin binds zinc. In *Streptococcus pneumoniae*, for example calprotectin reverses toxic sensitivity to zinc [190]. The zinc is bound by the solute-binding proteins of the ABC transport system. When calprotectin successfully chelates this divalent trace metal, however, the cells increase binding of Mn^++^, favoring growth and persistence [190]. Environment counts also, and the decreasing pH found in inflamed tissues reduces calprotectin heterotetramer formation and the chelation of trace metals [206]. By reducing the effectiveness of metal binding by calprotectin, solute binding proteins become more effective at capturing Mn^++^. Nutritional immunity mitigates against certain bacteria, including *S. pneumoniae* and *S. aureus* [190]. Indeed, many bacteria express solute-binding proteins as part of their ABC transporter systems, which bring nutritional solutes into the bacterial cell to aid cell growth. Many streptococci, including the oral species *S. gordonii* [207, 208], *S. mutans* [209], and other related species [210] have ABC transporter systems. In oral streptococci, calprotectin compromises the function of the ABC transporters and the utilization of trace metals, which negatively impacts growth [211]. Most microbes live in complex communities. More must be learned about how calprotectin affects commensal and pathogenic microbes existing in a complex community and whether sequestration of zinc or other trace metals might favor growth of commensal bacteria while limiting pathobionts and pathogens.

### Intracellular calprotectin

Calprotectin localizes after synthesis in the cytoplasm of cells including neutrophils, monocytes, and mucosal squamous epithelial cells [212, 213]. In neutrophil cytoplasm, calprotectin is the most abundant protein on a molar basis. Cytosolic calprotectin appears to directly protect the interior of squamous epithelial cells against invasive microbes by antimicrobial activity [36, 68, 80, 195] and indirectly by activating NADPH oxidases leading to production of reactive oxygen species (ROS) [214, 215]. After phagocytosis, neutrophils effect indirect calprotectin-dependent antimicrobial mechanisms [216, 217]. In neutrophils, calprotectin appears to act as a calcium relay to activate antibacterial ROS production [218], but direct antimicrobial activity by calprotectin in the cytosol of neutrophils has not yet been shown definitively.

In epithelial cells, calprotectin expression increases in response to microbes including *Fusobacterium nucleatum* and *P. gingivalis* [219, 220] and PAMPs such as lipopolysaccharide (LPS) [221] and flagellin [222]. Indeed, when bacteria bind, epithelial cells upregulate IL1α, which is released and then engaged by the IL1 receptor [82, 83]. Engagement of the IL-1 receptor signals through the p38-MAP kinase pathway, increasing CCAAT/enhancer binding protein β (C/EBPβ) transcriptional activity, which upregulates expression of calprotectin.

To counter intracellular calprotectin, bacterial species have evolved countermeasures. For example, *Listeria* induces calprotectin co-localization with the actin cytoskeleton and reduction in antimicrobial activity [68]. Fungi such as *Candida albicans* survive by promoting AMP effectors and efflux pumps and regulating downstream signaling pathways [45]. Encounters with phagocytosed bacteria such as *Porphyromonas gingivalis*, a prominent periodontal pathobiont, can also trigger the release of calprotectin from neutrophils into the extracellular environment [223]. Both monomers are vulnerable to oxidation on methionine and cysteine residues, which mitigates antimicrobial activity [169]. Clearly, the role of calprotectin in antibacterial defense depends on the cell source, multimerization state, which optimizes nutritional immunity, and cooperative mechanisms such as activation of ROS production, the cellular or pericellular localization, and the ability of the microbe to evade or subvert calprotectin.

## The antimicrobial effectiveness of calprotectin *in vivo*

*In vivo* studies can provide a deductive approach to understanding the contribution of calprotectin to innate immunity. In a study of experimental periodontitis in mice that compared wild type and S100A9^−/−^ (calprotectin^null^), expression of calprotectin minimized the emergence of a dysbiotic periodontal microflora, gingival inflammation, and periodontitis with loss of alveolar bone [159]. In wild type mice, the density of neutrophil infiltrate was lower and emergence of a dysbiotic microbial community was mitigated when compared to periodontitis in the calprotectin^null^ mouse. In the presence of calprotectin, the lower gingival inflammatory cell infiltrate could increase the alkaline pH environment, albeit with lower tissue Ca^++^ which is released from neutrophils. Inflammation is also generally accompanied by interstitial acidosis, which serves as another danger signal [224], and release of cellular calcium into the tissues [189]. In human periodontitis [164, 165], the neutrophil-rich inflammatory environment would also be expected to contain increased extracellular levels of calprotectin [191]. The elevation in calcium promotes tetramerization of calprotectin, increasing antimicrobial activity. In contrast, lower tissue pH would mitigate the ability of calprotectin to sequester Mn^++^ and tetramerize [206]. Tetramerization and stronger nutritional immunity in low tissue pH would, however, occur in the presence of higher Ca^++^ environments [190]. Given the complexity of inflammatory tissue environments, the effectiveness of antimicrobial calprotectin may be difficult to predict.

In wild type calprotectin-expressing mice, PMN-rich inflammation was dampened 2 days after ligature placement, whereas inflammation was robust in calprotectin^null^ mice [159]. At 5 days of ligature placement, inflammation was greater in the wild type mice. Hence, early inflammation was suppressed by calprotectin but over time inflammation was enhanced. The role of calprotectin appeared dichotomous over time. Eventually, extracellular soluble calprotectin heterodimer appears to serve as a proinflammatory *alarmin* by engaging TLR4 [171, 174, 176], RAGE [225–227], CD36 [184, 225], CD69 [186, 228], and perhaps other receptors. CD33, a highly conserved sialic-acid-binding immunoglobulin-like lectin (Siglec) expressed primarily on monocytes and myeloid progenitors [229], binds S100A9. Little is known, however, about engagement of other forms of calprotectin [18]. Signaling through these receptors activates proinflammatory signaling pathways (Table 1, Figure 2). Receptor engagement by calprotectin is cell type and tissue specific. Human oral epithelial cells and gingival fibroblasts express TLR4; human oral epithelial cells also express TLR2 and RAGE which, like TLR4, interact with the calprotectin heterodimer [177].

Calprotectin stimulates murine neutrophils to migrate and adhere to integrin substrates [183]. Loss of calprotectin in mouse models make neutrophils less sensitive to chemotactic agents including IL-8 [230]. The N1 neutrophil phenotype is more proinflammatory than the N2 phenotype. The N1 neutrophils respond to calprotectin (S100A9) by chemotaxis and elevated expression of NADPH-oxidase [231]. Hence, the response to calprotectin by neutrophils will depend on the prevalence of N1 and N2 phenotypes *in vivo*. In reported infection models to date, the predominance of N1 or N2 neutrophil phenotypes is generally not reported. For example, in a murine model of *S. pneumoniae*-induced lung infection, calprotectin is required for the appearance of a robust neutrophil infiltrate with the regulated production of G-CSF and a reduction of the infectious load in the lungs [232]. Whether calprotectin acts dichotomously as an alarmin or as an inflammation-dampening protein complex is strongly suggested to depend on the prevalence of a dominant N1 phenotype in the response to infection.

Calprotectin expression in human infants (and newborn mice) sustains the composition of the healthy developing gut microbiome [233]. Infants with high fecal concentrations of calprotectin had greater abundance of Actinobacteria and Bifidobacteriaceae and fewer Gammaproteobacteria including opportunistic Enterobacteriaceae. Low calprotectin expression was associated with sepsis and obesity by 2 years of age [233].

Stability of the human infant gut microbiome is supported by breast milk, which contains concentrations of calprotectin that are six-times higher than in the serum of newborns [234]. In infants, calprotectin is anti-inflammatory. After term vaginal delivery, calprotectin levels in breast milk are higher than after pre-term and Caesarian section deliveries. Two weeks after birth, breast milk calprotectin decreases to the levels in adult sera. At these levels, breast milk calprotectin significantly inhibited growth of Mn^++^-sensitive bacteria such as *Staphylococcus aureus* and group B streptococci (neonatal sepsis related bacteria) [234]. After term deliveries, higher levels of calcium in breast milk were associated with a reduction in the amount of calprotectin needed to inhibit pathogens, presumably due to heterotetramer formation. For comparison, in S100A9^−/−^ mice, GI Enterobacteriaceae were more abundant when compared to wild-type mice. Simulating neonates with low levels of calprotectin, S100A9^−/−^ mice also showed altered intestinal macrophage phenotypes and reduced numbers of T-regulatory cells [234]. These characteristics may predispose human and murine neonates to sepsis. Neonatal sepsis may be prevented or mitigated by the high concentration of calprotectin in breast milk and protect against lactational mastitis in the mother.

## AMPs in the oral cavity

The oral cavity contains an estimated 700 microbial species that organize into communities reflecting the unique anatomic and nutritional features of the local habitats [235]. Hence, the communities residing on the tongue differ from those on the smooth enamel surfaces, which differ from those in the gingival crevice or within gingival epithelial cells. The microbes recovered in saliva cannot be viewed as a resident community since saliva in the mouth replenishes continually, with about 1 mL secreted and swallowed each minute by healthy adults. The salivary microbiome reflects the organisms shed from the oral surfaces on an ongoing basis [236] and can change due to underlying conditions such as inflammatory bowel disease [237].

In the oral cavity, AMPs produced in different anatomic sites are among the factors that control the growth and complexity of resident microbes (Table 2). There are no fewer than 18 discrete molecular families that confer antimicrobial control in the oral cavity. The relative abundance of different AMPs in intraoral sites serves as an important ecological determinant that contributes to the abundance, organization, complexity, and stability of the resident microbial communities [99, 135, 159, 267, 268]. At the epithelial interfaces, each AMP and cooperation between them are poorly understood as influences of the composition and pathogenicity of the oral communities.

**Table 2:** Antimicrobial proteins in the oral cavity.

**AMPs**	**Roles**	**References**
α-Amylase	Binds adhesins to block surface binding Inhibits bacterial growth Conversely, may aid biofilm formation	[238]
Adrenomedullin	Found in gingival crevicular fluid and saliva cationic peptide creates membrane pores, cell leakage and rupture	[239]
Antibodies Secretory IgA Salivary IgG	Bind bacteria, fungi, and viruses Agglutination and clearance Inhibition of binding Promote phagocytosis, degranulation, and/or cytokine production by immune cells	[240]
Azurcidin (CAP37; heparin binding protein, HBP)	Antibacterial, Gram-negative preference Chemoattractant and activator of monocytes, macrophages, and T lymphocytes	[241, 242]
Bactericidal/permeability increasing protein (BPI); BPI-like, salivary PLUNC proteins	LPS-binding N-terminal domain bactericidal, endotoxin neutralizing C-terminal domain opsonic, binds phagocytes BPI and short type S-PLUNC have N-terminal domains, presents Gram-negative bacteria and LPS-rich particles Long type L-PLUNC has both the N-terminal LPS-binding domain and the C-terminal opsonic domain Parotid secretory protein (PSP) binds surface LPS to promote agglutination	[243]
Calprotectin	Found in saliva, gingival crevicular fluid, oral mucosal epithelial cells Antibacterial and antifungal Sequesters essential trace metals from microbes leading to nutritional immunity Functions intra- and extracellularly	[36, 67, 80, 160, 162, 164–167, 169, 177, 197, 211, 219]
Cathelicidins (LL-37)	18 kDa cationic peptide, when cleaved releases smaller peptides (including LL-37) of higher antimicrobial activity Mode of action: cationic peptide aggregation on bacterial membranes resulting in pore formation, membrane leakage, and rupture Immunomodulatory agonist of certain cell membrane receptors	[244]
C-C motif chemokine 28 (CCL28)	Antimicrobial activity against Gram-positive, Gram-negative bacteria, and fungi Causes membrane permeability As a chemokine, regulates chemotaxis of cells that express CCR3 and CCR10	[245, 246]
Cystatins	Cysteine protease inhibitors Immunomodulatory properties Antagonizes *P. gingivalis*	[247]
Defensins	Present in saliva and expressed by cells and tissues Broad spectrum antibacterial and antifungal Immunomodulatory properties	
Histatins	Broad range fungicidal Most effective against yeast, *C. albicans* Non-lytic release of ATP leading to cell death Formation of oxygen radicals Most abundant in oral cavity	[248–250]
Lactoferrin/lactotransferrin	Active against bacteria, fungi, and viruses Potent iron chelator, sequesters free iron resulting in antimicrobial, and bacteriostatic effects Binds and damages bacterial membranes Binds viral particles and receptors Proteinase inhibitor, anti-virulence factor	[251–253]
Lysozyme	Antibacterial; muramidase activity weakens bacterial cell wall leading to lysis Aggregates bacteria promoting clearance	[254–256]
	Activates bacterial autolysins which destroy cell walls Anti-fungal and antiviral activity	
Salivary Mucins	Promotes bacterial agglutination MUC5B and MUC7 protect host cells from viral entry	[257]
Neuropeptides	Direct antimicrobial activity against oral pathogens and fungi	[258, 259]
Peroxidases (lactoperoxidase and myeloperoxidase)	Catalyze the oxidation of thiocyanate in the presence of hydrogen peroxide creating a bactericidal hypothiocyanite (OSCN^−^)	[260]
Deleted in malignant brain tumor 1 (DMBT1/DMBT1, gp340) Salivary agglutinin DMBT1(SAG)	Aggregates and clears *S. mutans, S sanguinis* and influenza A virus from oral cavity Prevents binding of *C. albicans* to receptors surface absorbed SAG: role in complement activation	[261–263]
Secretory leukocyte protease inhibitor	Present in saliva, produced by oral keratinocytes Serine protease inhibitor Cationic peptide, creates membrane pores, membrane leakage, and rupture Antibacterial, antifungal, and antiviral	[264–266]

## Use of mouse genetic models and the development of AMP therapeutics

Use of mouse models to identify candidate genes has proven invaluable in increasing our understanding of many diseases. As we illustrate, a gene of interest can be shown to contribute to a functional or disease phenotype. Typically, however, the results show only that the gene product is necessary but not necessarily sufficient or the sole protein of interest. In the case of studying AMPs and innate immunity, we see functional redundancy in the control of the microbiota. We can also be confounded by interactions between AMPs and immune cells that contribute to an observed phenotype. AMPs can contribute to the development of the murine immune system and sustain the development of a healthy microbiome. As investigators translate data about a protein/peptide into a treatment, results in the human clinical trials may not mirror the potency or effectiveness predicted by genetic data in mice. To identify the cells and tissues responsible for the AMP activity, the use of conditional knockout strains may be useful when expression is under control of cell and tissue-specific promoters. Knock-in studies that add or increase expression of a specific gene would make the experimental paradigm more robust. Bacteria and fungi are generally sensitive to AMPs including calprotectin and have co-evolved successfully, but resistance mechanisms in key pathogens have been characterized [269–273]. Engineered AMPs have been suggested as therapeutics to compliment or replace the use of antibiotics [274] where resistance is a major therapeutic problem [275–278]. To replace or serve as an adjunct to antibiotics, specific AMP therapeutics can be developed in animal models. One interesting strategy would target production by the native AMP-producing cells. Indeed, we may be able to develop AMP therapeutics that target the mucosal barrier epithelium where infections originate.

## Conclusions

The contribution of AMPs to antimicrobial immunity is a crucial first defense of the epithelial barrier. The AMPs differ in structure and include the evolutionarily conserved families of pore-forming peptides and the essential trace metal binding proteins that create antimicrobial nutritional immunity. Functioning in affected tissues, AMPs serve cooperatively to thwart bacterial, fungal, and viral infections. In epithelial tissues spaces, the AMPs can present in microbe-trapping antimicrobial NETs to circumscribe and neutralize invading pathogens. Functioning intracellularly, AMPs protect cells against invasive microbes. Given their presence in different anatomic sites in the oral cavity, AMPs appear to confer site-specific microbial control. During the immune response to infection, parsing out the role of the AMPs even when mutated or deleted in genetic models can be challenging. Each AMP functions in the constellation of related proteins and peptides and adaptive immunity. Furthermore, crosstalk between systems can upregulate compensatory mechanisms. Nonetheless the evolution of AMPs in lower species indicates that innate immunity fortifies the epithelial barrier in the absence of the complex immunity common to humans and other mammals. Given that AMPs have broad-spectrum antimicrobial activity and do not readily promote microbial resistance, translational development of AMPs as therapies appears warranted in the face of growing microbial resistance to conventional antibiotics. Indeed, AMPs may be applied therapeutically to fortify the oral epithelial barrier against infection.

## Author contributions

KJ and MH created the concept and drafted this manuscript. KJ created the figures using BioRender^©^. MH finalized the manuscript. All authors contributed to the article and approved the submitted version.

## Funding

Investigations about the structure and function of AMPs in the authors' laboratory were supported by R21DE025711, R01DE021206, and RO1DE11831.

## Conflict of interest

The authors declare that the research was conducted in the absence of any commercial or financial relationships that could be construed as a potential conflict of interest.

## Publisher's note

All claims expressed in this article are solely those of the authors and do not necessarily represent those of their affiliated organizations, or those of the publisher, the editors and the reviewers. Any product that may be evaluated in this article, or claim that may be made by its manufacturer, is not guaranteed or endorsed by the publisher.
